# Caprine Endometrial Mesenchymal Stromal Stem Cell: Multilineage Potential, Characterization, and Growth Kinetics in Breeding and Anestrous Stages

**DOI:** 10.1155/2017/5052801

**Published:** 2017-03-05

**Authors:** Amin Tamadon, Davood Mehrabani, Younes Zarezadeh, Farhad Rahmanifar, Mehdi Dianatpour, Shahrokh Zare

**Affiliations:** ^1^Stem Cell Technology Research Center, Shiraz University of Medical Sciences, Shiraz, Iran; ^2^School of Veterinary Medicine, Shiraz University, Shiraz, Iran; ^3^Department of Basic Sciences, School of Veterinary Medicine, Shiraz University, Shiraz, Iran; ^4^Department of Human Genetics, School of Medicine, Shiraz University of Medical Sciences, Shiraz, Iran

## Abstract

The endometrial layer of the uterus contains a population of cells with similar characteristics of mesenchymal stem cells (MSCs). In the present study, caprine endometrial mesenchymal stromal stem cells (En-MSCs) characters and differentiation potential to chondrogenic, osteogenic, and adipogenic cell lines as well as their growth kinetics in breeding and anestrous stages were evaluated. En-MSCs were enzymatically isolated from endometrial layer of the uterus of adult goats and were cultured and subcultured until passage 4. The growth kinetics and population doubling time (PDT) of caprine En-MSCs in breeding and anestrous stages were determined. En-MSCs in passage 4 were used for the karyotyping and differentiation into chondrocytes, osteocytes, and adipocytes. The PDT in anestrus phase was 40.6 h and in cyclic goats was 53 h. En-MSCs were fibroblast-like in all passages. The number of chromosomes was normal (2*n* = 60) with no chromosomal instability. Chondrogenic, osteogenic, and adipogenic differentiation of En-MSCs was confirmed by staining with Alcian blue, Alizarin red, and Oil Red O, respectively. Caprine En-MSCs demonstrated to be an alternative source of MSCs for cell therapy purposes in regenerative medicine.

## 1. Introduction

Friedenstein et al. [1] were the first who reported isolation of mesenchymal stem cells (MSCs) from bone marrow. After that adipose tissue [2], umbilical cord blood [3], and endometrium [4] were other reported tissues as sources of MSCs. MSCs were shown to be undifferentiated clonogenic cells with self-renewal and multilineage differentiation properties [5]. Their uses in regenerative medicine are emerging and can be an alternative choice to promote functional recovery in patients suffering from various diseases that may be the cause of death and permanent disability [6]. Multilineage properties of MSCs were demonstrated to be dependent on the source and the donor [7].

As a large animal model and similarity of human knee joint, goat is widely investigated for bone tissue engineering [8]. Caprine MSCs have been used in tissue engineering for cartilage regeneration and restoring articular cartilage due to the chondrogenic potential of MSCs [9, 10]. Genetic engineering of caprine MSCs also has been done for cell therapy by enhancing differentiation potential of MSCs [11], tracking of transplanted cells using green fluorescent protein [12], and facilitating tissue generation process [13].

Information on the goat MSCs characterization is relatively more recent and it is little compared to investigations in commonly used species such as the human and mouse. The bone marrow and adipose tissue are the reported sources of access to MSCs in goats [14]. Furthermore, there are relatively reports on goat MSCs which are isolated from umbilical cord [15, 16].

Another source of MSCs is endometrial layer of the uterus [4]. The endometrium of the uterus in mammals is capable of high and periodic self-renewal regeneration. Also, reconstruction of the endometrium after parturition and rapid proliferation and restoration indicates that the endometrium can be a rich source of MSCs [17]. On the other hand, larger amounts of MSCs are needed for use in clinical and experimental applications than the starting population isolated MSCs which must be expanded* in vitro*. Furthermore, increase in doubling time [18] and cellular senescence [19] and loss in multipotentiality [20] and changes in cell surface marker profile [21] are the results of increasing number of passages of long term* ex vivo* culture of MSCs. Regarding these subjects, endometrial mesenchymal stromal stem cells (En-MSCs) may be a more appropriate source in comparison with bone marrow [4]. Therefore, in the present study, the method of isolation, characterization, and multilineage differentiation potential of En-MSCs as well as comparison of the growth kinetics of En-MSCs in both anestrus and cyclic goats were evaluated.

## 2. Materials and Methods

All procedures are in accordance with animal guideline care of Ethical Committee of Shiraz University. All applicable institutional and national guidelines for the care and use of animals were also followed.

### 2.1. Uterine Tissue Sampling

Uteri were collected from the healthy mature nonpregnant Fars native nanny goats with 2 to 3 years of age in breeding (February) and anestrous (June) stages from Shiraz Slaughter House in Shiraz, southern Iran. Breeding season of Fars native goats starts from late summer by decrease of daylight to darkness ratio and anestrus stage lasts from April to August when daylight to darkness ratio increases [22]. Furthermore, presence or absence of corpus luteum on ovaries of mature goats was selected as another index of breeding or anestrous stages, respectively [23]. Uteri were transferred in sterile plastic bags in the presence of ice (1°C) to Stem Cell Laboratory of Stem Cell and Transgenic Technology Research Center, Shiraz University of Medical Sciences, Shiraz, Iran.

### 2.2. En-MSCs Isolation and Culture

**Figure 1:**
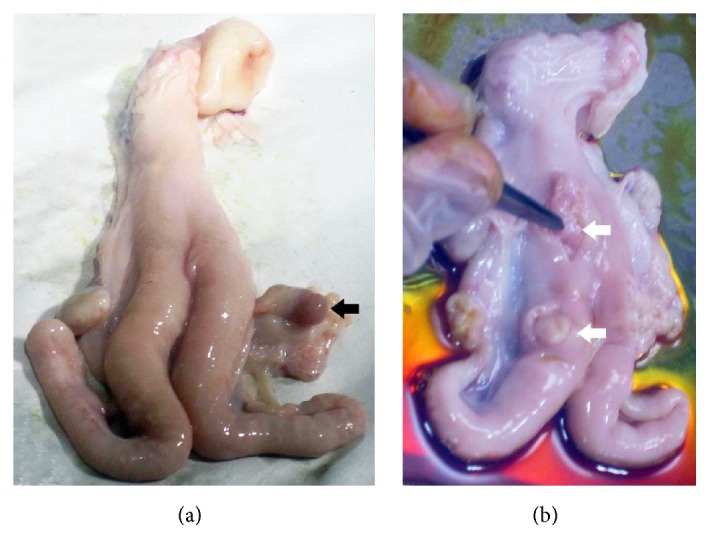
Uteri of goats (a) in breeding stage with corpus luteum (black arrow) and (b) in anestrus stage. The locations of sampling of endometrial tissues are shown by white arrows on body and horn of the uterus.

After removing of excess fat tissue surrounding the uterus, the uterine surfaces were rinsed well with warmed sterile normal saline. Under laminar flow (Class II, Jal Tajhiz, Iran) in sterile condition, six full-thickness samples of endometrial tissue (10 × 10 × 5 mm^3^), four on both horns and two on the uterine body from both cotyledonary and intercotyledonary regions, were collected using sterile (autoclaved) surgical instruments (Figure 1). Samples of each uterus were suspended separately in 15 mL falcons containing Dulbecco's Modified Eagle's Medium (DMEM, BioWest, France) supplemented with 10% fetal bovine serum (FBS, BioIdea, Iran) and 1% penicillin/streptomycin (BioWest, France). The falcons were kept at 4°C for 24 h. Then, fine slices of endometrial tissue samples were placed in Hank's balanced salt solution (HBSS, BioWest, France) containing collagenase 0.1% type A1 (Invitrogen, France) in a humid-incubator with 5% CO_2_ and was incubated at 37°C for 1 h and then the obtained cell suspension was passed through a 150 *μ*m filter and the cells were separated by centrifugation (5 min, 1200 rpm).

The isolated cells were transferred to DMEM/F12 medium containing 10% FBS and incubated at 37°C and 5% CO_2_ and non-MSCs in primary culture were suspended on the medium and, by changing medium, they were removed. En-MSCs were attached to the bottom of the T25 culture flasks. Changing medium was done every three days and the cells after reaching 70–80% confluency were isolated by trypsin/EDTA (0.5%, Sigma, USA) and passaged. The cells were cultured for 4 passages.

### 2.3. En-MSCs Growth Kinetic

In order to have a cell count, the cells were mixed with the equal volume of cell suspension and trypan blue and were stained, while the cells were later counted by hemocytometer. By seeding the cells, growth curve and population doubling time (PDT) were provided and calculated as previously described [4]. In details, for the assessment of growth characteristics, En-MSCs at passage 4 from both groups were separately seeded in 12-well plates at a density of approximately 2.2 × 10^4^ cells per well in triplicate. Furthermore to compare the effect of plate bottom area on cell growth En-MSCs of anestrous goats were seeded at a density of approximately 2.2 × 10^4^ cells per well in 12-well and 24-well plates. Cells were collected from each well 1 to 7 days after seeding and counted microscopically to produce cell growth curves. The curves were drawn using GraphPad Prism (Version 5.01; GraphPad software Inc., San Diego, CA, USA). To compare the* in vitro* proliferation rate, the PDT value was determined for each group of cells. PDT was calculated using the formula PDT = *T*ln⁡2/ln⁡(*X*_*e*_/*X*_*b*_), in which *T* is the incubation time in hours, *X*_*b*_ represents the cell number at the beginning of the incubation time, and *X*_*e*_ corresponds to the cell number at the end of incubation time.

### 2.4. Freezing and Thawing

After counting the cells in each passage and recording them, the cells were divided into two parts, one part for transferring into the next passage and the other for freezing to evaluate their survival after thawing. In order to freeze the cells, the freezing medium containing 50% FBS, 40% DMEM, and 10% dimethyl sulphoxide (DMSO, MP Bio, USA) was used. The cells suspended in freezing medium were transferred to a freezer (−20°C) immediately for 1 h and then to another freezer (−70°C) for 24 h and finally into liquid nitrogen tank (Atocel, Austria) for long-term storage. To evaluate their survival after thawing, 10 days later, the cryotubes were removed from the liquid nitrogen and warmed in a 37°C water bath rapidly and were immediately transferred into a DMEM culture medium. They were centrifuged at 1200 rpm for 7 min and the cell pellet was suspended into DMEM culture medium. After thawing, the viability was evaluated by the trypan blue exclusion test (0.4% trypan blue in phosphate buffered saline, PBS). Cell viability was calculated as the number of viable cells divided by the total number of cells and expressed as a percentage.

### 2.5. Karyotyping

Karyotype of adult goat cells from passage 4 was prepared. For this purpose, the cells were incubated for 2 h and 30 min in the vicinity colcemid. KCL (0.075 M) was used as a hypotonic solution. The cells were stabilized by methanol and acetic acid at a ratio of 3 : 1 and 2 : 1 and Giemsa staining was used to stained chromosomes. The internationally accepted cytogenetic system of nomenclature (ISCN: International System of Cytogenetic Nomenclature) is used for classification of the chromosomes.

### 2.6. Chondrogenic Differentiation of En-MSCs

A sample of cells from passage 4 was used. The 9-well plate was used where 6 wells were considered to differentiate into chondrocytes and 3 wells as control. The wells of the control group received just DMEM/F12 medium and were cultured. But, in the wells of differentiation group, chondrocytes differentiation medium was added. Briefly, 5 × 10^4^ cells in 15 *μ*L medium droplets were placed at the center of each well of the 9-well plate. The plate was put in an incubator for 2 h to provide cell attachment. Then, 500 *μ*L of premade chondrogenic differentiation medium (StemPro Chondrogenesis Differentiation Kit, Gibco, UK) was added. The medium was changed every other day for 21 days to form cartilage micromass. After 21 days, the cells were fixed with 95% methanol and then were stained with 0.1% Alcian blue 8GS (Fluka, Buchs, Switzerland) in 0.1 M HCL overnight.

### 2.7. Osteogenic Differentiation of En-MSCs

To evaluate the osteogenic differentiation, En-MSCs were seeded into a 9-well plate. At 80% confluency, the cells were cultured for 21 days with low glucose DMEM containing 100 nM dexamethasone (Sigma, USA), 0.05l M ascorbate-2-phosphate (Wako Chemicals, USA), 10 mM b-glycerophosphate (Sigma, USA), 1% penicillin/streptomycin, and 10% FBS. The medium was replaced every other day. After 21 days, osteogenic differentiation was determined using Alizarin red staining (Sigma, USA).

### 2.8. Adipogenic Differentiation of En-MSCs

For adipogenic differentiation, En-MSCs were seeded in a 9-well plate. When they reached 80% confluency, they were induced to adipogenic differentiation with adipogenic induction medium containing DMEM low glucose, 10% FBS, 0.5 mM isobutyl-methylxanthine (Sigma-Aldrich), 10% FBS, 0.5 mM isobutyl-methylxanthine (Sigma-Aldrich), 1 *μ*M dexamethasone, 10 *μ*M insulin, and 200 *μ*M indomethacin (Sigma-Aldrich). The plates were maintained for 21 days and medium was replaced every other day. At the end of period, the cultures were fixed by 10% formalin solution for 10 minutes. Fixed cells were subjected to Oil Red O (Sigma-Aldrich), which specifically stains lipid droplets.

### 2.9. Statistical Analysis

The mean and SE of counted cells in growth curve analysis were compared using independent sample *t*-test (SPSS software, version 11.5, SPSS Inc, Chicago, USA). *P* ≤ 0.05 was considered statistically significant.

## 3. Results

### 3.1. En-MSCs Morphology

The caprine endometrium differed from human in that it had glandular intercaruncles and aglandular caruncles. To evaluate anestrus and breeding uteri, cells were isolated from the whole caprine endometrium including both caruncle and intercaruncle areas.

**Figure 2:**
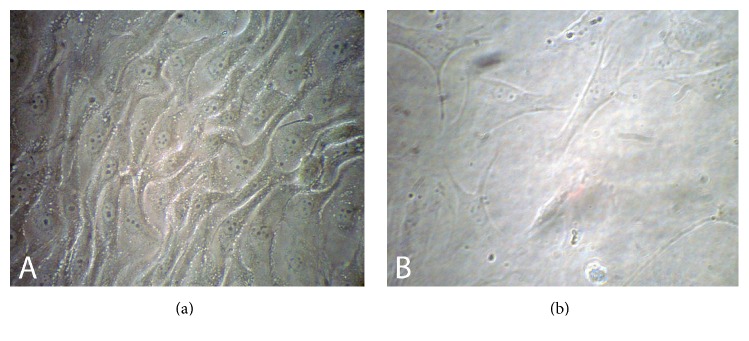
Fibroblast-like morphology of caprine endometrial mesenchymal stromal stem cells in (a) anestrous and (b) breeding stages (magnification: ×200).

During the primary cell culture, cells were observed with inverted microscope and after 24 h, the number of cells attached to the bottom of the flasks, and their shape was fibroblast-like and several colonies were found at the bottom of culture flasks. Two groups of colonized cells with different morphology were visible in primary culture. Some colonies were compact and round which were similar to endometrial epithelial cells colony and the others were stromal cells. After four days of culturing cells, it was found that the cells grew and the culture flask was full of the single layer of cells. After the first passage, most of epithelial cells were not detached from the primary culture flasks and the adhesion cells in subcultured flasks had formed the population of uniform fibroblast-like cells (Figure 2).

### 3.2. En-MSCs Growth Kinetics

**Figure 3:**
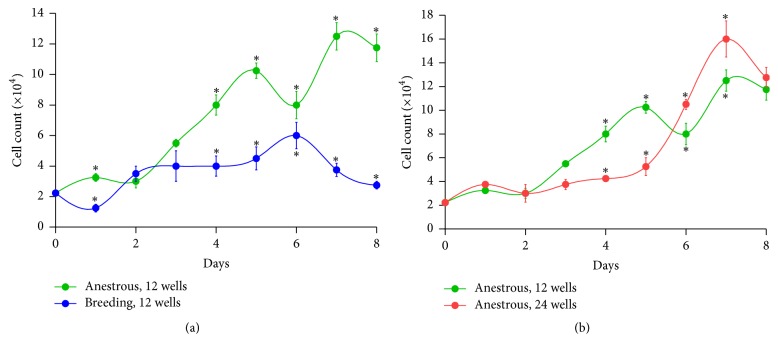
Comparison of growth curves of goat endometrial mesenchymal stromal stem cells after seeding of 2.2 × 10^4^ cells (a) in 12-well culture plates between anestrous and breeding stage and (b) between 12-well and 24-well culture plates in anestrus stage.

In anestrous goats, when 2.2 × 10^4^ En-MSCs were seeded into 12-well culture plates at the starting point, the growth of En-MSCs resulted in a PDT of 40.6 h. While, in cyclic goats using 12-well culture plates, PDT period increased significantly into 53 h. However, by seeding the same number of anestrus goat cells into 24-well culture plates, this PDT reached 49.6 h. Comparing the growth kinetic of En-MSCs of goats in breeding and anestrus stage which their cells seeded in 12-well plates, a rise in number of cells at the log phase and a shorter PDT was shown in anestrus stage in comparison with breeding stage (Figure 3(a)). Moreover, comparing the 12-well and 24-well culture plates and the growth kinetic of En-MSCs of anestrus goats, it was shown that, in the larger space with the same cell number, growth in the 12-well plate resulted in an increase in proliferation of cells at the log phase and a shorter PDT in comparison with the 24-well plate (Figure 3(b)).

### 3.3. Karyotype Analysis

**Figure 4:**
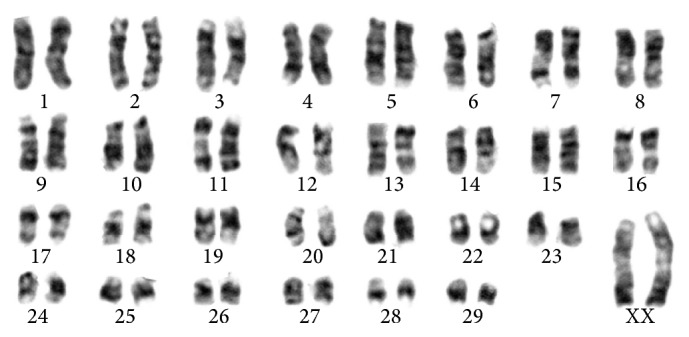
Karyotype of endometrial mesenchymal stromal stem cells in passage 4 of cyclic goats.

Fifty metaphase cells from passage 4 from the cyclic adult goat sample were analyzed. The number of metaphase chromosomes in all cells was 60 normal acrocentric chromosomes (Figure 4). More than 98% of metaphases were diploid.

### 3.4. Differentiation into Chondrocytes, Osteocytes, and Adipocytes

**Figure 5:**
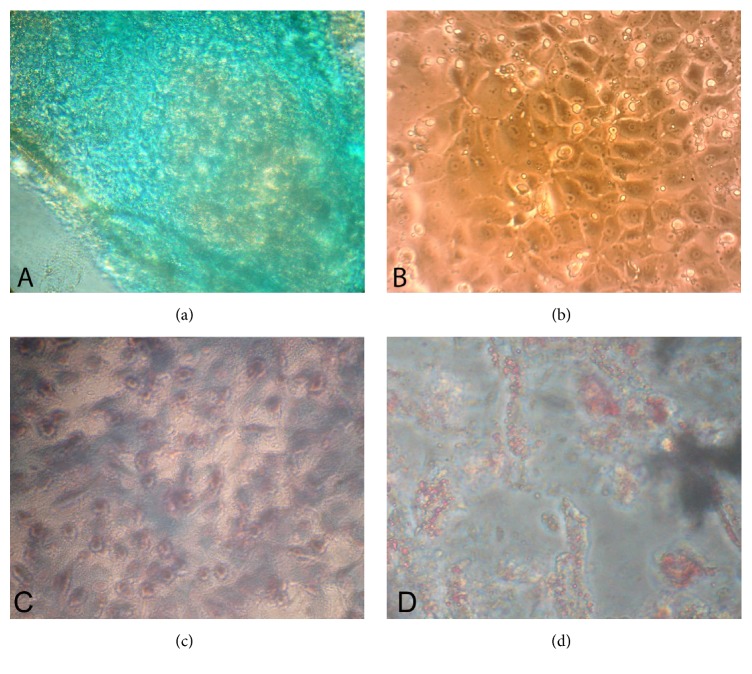
Differentiation of endometrial mesenchymal stromal stem cells (En-MSCs) into (a) chondrocytes stained with Alcian blue shows methachromatic appearance (magnification: ×40). (b) Control cells that remained colorless (magnification: ×100). (c) Alizarin red staining revealed calcification and osteogenic differentiation of En-MSCs in goat (magnification: ×100). (d) Oil Red O staining revealed lipid droplets and adipogenic differentiation of En-MSCs in goat (magnification: ×40).

After 24 hours, before Alcian blue staining, undifferentiated cells in observed wells were round and dense concentric circular colonies were observed. After staining, extracellular matrix was taken to dark blue stain where the so-called Alcian blue stain had metachromatic appearance to extracellular matrix (Figure 5(a)). The metachromatic appearance matrix confirmed differentiation of goat endometrial stem cells into chondrocytes. This appearance was observed because of proteoglycans and glycosaminoglycans in the extracellular matrix, the two substances made by chondrocytes in the extracellular matrix. The control group cells were fibroblast-like in shape with mesenchymal properties that were not differentiated into chondrocytes. Colonies formed by control group had lower density compared to differentiated group. Mesenchymal colonies were irregular and polygonal. Control cells were not formed in extracellular matrix and, therefore, the cellular masses of control group were completely colorless using Alcian blue stain (Figure 5(b)).

En-MSCs also successfully differentiated into adipocytes and osteoblasts lineages (Figures 5(c) and 5(d)). After three weeks, staining of differentiated caprine En-MSCs in osteogenic media into osteocytes with Alizarin red resulted in presence of calcium deposits and demonstrated mineralized matrix. Adipogenesis of En-MSCs was detected by the formation of lipid droplets stained with Oil Red O staining, 21 days after induction. The negative control (noninduced) cells for each type of differentiation were negative for Alizarin Red and Oil Red O stains.

## 4. Discussion

Due to the diverse and inclusive use of MSCs in cell therapy, investigations focused on the isolation of these cells from different sources and on the characteristics of them [24]. In this research, after isolation of the cells, characters of caprine En-MSCs including self-renewal, morphology, karyotyping, freezing, and thawing effects and multilineage differentiation potential were evaluated. This is the first study to characterize En-MSCs in the caprine endometrium. Consistent with our findings, recently, Letouzey et al. [25] showed that the ovine endometrium contains a small subpopulation of MSCs. Caprine En-MSCs had several relevant MSCs criteria including clonogenicity and multilineage mesodermal differentiation. These criteria also were shown in heifer En-MSCs [4].

The importance of determination of self-renewal and growth behaviors of the caprine En-MSCs is on application of these cells in cell therapy which needs appropriate number of these cells after three or four passages. Our findings showed, in anestrus stage, a higher number of cells at the log phase and with a shorter PDT grown in comparison with breeding stage. It is shown that En-MSCs as stromal cells require several growth factors for colony-forming cells/units activity including epidermal growth factor (EGF), platelet-derived growth factor BB (PDGF-BB), transforming growth factor *α* (TGF*α*), and basic fibroblast growth factor (bFGF) [26]. Interestingly, intraluminal treatment with EGF gradually reduced uterine activity of diestrus goats [27]. It can be speculated that increase of En-MSCs growth during anestrus may be related to the increase and accumulation of growth factors in endometrial tissue in comparison with cyclic stages.

After cell expansion, En-MSCs fibroblastic morphology is the most important qualitative parameter evaluated by light microscopy in a regular pattern. In this study, 24 h after the primary culture, some cells were attached to the flasks and morphology of cells was changed to fibroblast-like shape. After four passages, they also kept this fibroblast-like morphology. En-MSCs had high proliferative properties and colony forming unit (CFU) activity. MSCs are defined as plastic adherent cells [6]. Adherent property identical to the MSCs seen in other adult tissues was also noticed in caprine En-MSCs. This morphology referred to the mesenchymal property of these cells isolated from goat endometrium which has been confirmed in the En-MSCs of sheep [25], heifer [4], and human [28].

Isolated En-MSCs of the goat endometrium were differentiated to chondrocytes, osteocytes, and adipocytes, and this differentiation was confirmed by staining with the Alcian blue, Alizarin red, and Oil Red O, respectively. This research result was similar to the research conducted by Wolff et al. [29] on the differentiation potential of human En-MSCs showing their multipotency and their potential to differentiate into chondrocytes, osteocytes, and adipocytes* in vitro*. In the other studies, the differentiation of these cells to adipocytes in heifer [4] and osteocytes in cow [30] and human [31] had been confirmed. Recently, adipogenic, chondrogenic, osteogenic, and myogenic properties of ovine En-MSCs also had been shown [25]. Therefore, according to this character of caprine En-MSCs, these cells can be applied in translational studies on bone or cartilage regenerative medicine in this animal model.

In this study, result of En-MSCs karyotype showed that number of chromosomes was 2*n* = 60 and all goat samples were normal and stably diploid. Fifty-eight autosomal chromosomes and two sex chromosomes (XX) were observed as reported before [32]. Homologous chromosomes were quite similar in shape and structure, and no difference was observed in the form of homologous. Karyotype analysis showed that En-MSCs derived from caprine endometrium, after 4 passages, were quite normal in karyotype and with no chromosomal abnormalities such as duplications or deletions in any part of chromosome.

In conclusion, the present study showed that caprine En-MSCs were morphologically similar to MSCs while their differentiation potential was also confirmed. The PDT of these cells revealed that anestrus goats could yield more cells in a shorter period of time. Therefore, according to normal karyotype of these cells and their multilineage differentiation potential properties, En-MSCs can open a new window in stem cell therapies and as a readily available source in regenerative medicine investigations on animal models.
